# Unexpected visible light driven photocatalytic activity without cocatalysts and sacrificial reagents from a (GaN)_1–*x*_(ZnO)_*x*_ solid solution synthesized at high pressure over the entire composition range†

**DOI:** 10.1039/c7ra08509e

**Published:** 2018-02-27

**Authors:** H. A. Naveen Dharmagunawardhane, Alwin James, Qiyuan Wu, William R. Woerner, Robert M. Palomino, Alexandra Sinclair, Alexander Orlov, John B. Parise

**Affiliations:** Department of Materials Science and Engineering, Stony Brook University Stony Brook New York 11794 USA; Department of Chemistry, Stony Brook University Stony Brook New York 11794 USA alwin.james@stonybrook.edu john.parise@stonybrook.edu; Department of Geosciences, Stony Brook University Stony Brook New York 11794 USA; Brookhaven National Laboratory, Chemistry Department Bldg 555 Upton NY 11973 USA; Mineral Physics Institute, Stony Brook University Stony Brook New York 11794 USA; Joint Photon Sciences Institute, Stony Brook University Stony Brook New York 11794 USA

## Abstract

Optical and photocatalytic properties were determined for the solid solution series (GaN)_1–*x*_(ZnO)_*x*_ synthesized at high pressure over the entire compositional range (*x* = 0.07 to 0.9). We report for the first time photocatalytic H_2_ evolution activity from water for (GaN)_1–*x*_(ZnO)_*x*_ without cocatalysts, pH modifiers and sacrificial reagents. Syntheses were carried out by reacting GaN and ZnO in appropriate amounts at temperatures ranging from 1150 to 1200 °C, and at a pressure of 1 GPa. ZnGa_2_O_4_ was observed as a second phase, with the amount decreasing from 12.8 wt% at *x* = 0.07 to ∼0.5 wt% at *x* = 0.9. The smallest band gap of 2.65 eV and the largest average photocatalytic H_2_ evolution rate of 2.31 μmol h^−1^ were observed at *x* = 0.51. Samples with *x* = 0.07, 0.24 and 0.76 have band gaps of 2.89 eV, 2.78 eV and 2.83 eV, and average hydrogen evolution rates of 1.8 μmol h^−1^, 0.55 μmol h^−1^ and 0.48 μmol h^−1^, respectively. The sample with *x* = 0.9 has a band gap of 2.82 eV, but did not evolve hydrogen. An extended photocatalytic test showed considerable reduction of activity over 20 hours.

## Abstract 2

(GaN)_1–*x*_(ZnO)_*x*_ synthesized at high pressure produces H_2_ in the presence of visible light without any cocatalysts or sacrificial reagents.

## Graphical abstract

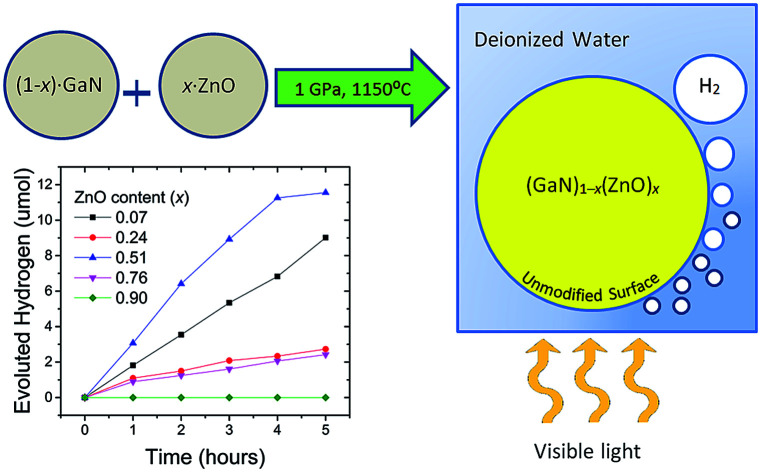

## Introduction

The world's energy needs are estimated to double by the middle of the twenty-first century.^1^ Without significant advances in alternative energy sources, this will raise the demand for fossil fuels for electricity production resulting in increases in CO_2_ emissions, which will contribute to global warming and irreversible environmental damage.^1^ Hydrogen, a clean, storable and transportable alternative to fossil fuels, can be produced, with minimal environmental impact, *via* overall photocatalytic water splitting.

Photocatalytic water splitting was first demonstrated using a TiO_2_ semiconductor anode and Pt cathode by Honda and Fujishima in 1972.^2^ Oxide photocatalysts, although known to spilt water under UV light, usually have band gaps above 3.0 eV, and cannot efficiently utilize the abundant visible light component of solar radiation.^3^ On the other hand, oxynitride compounds of d^0^ and d^10^ cations tend to have an appropriate band structure for visible light absorption and water splitting due to the contribution of N 2p atomic orbitals, which raises the valence band edge energy, and thereby decreasing the overall band gap.^4^

Maeda *et al.* discovered that the solid solution of wurtzite type GaN and ZnO can both absorb light in the visible region and perform overall water splitting with the presence of cocatalysts.^5^ The members of the solid solution series (GaN)_1–*x*_(ZnO)_*x*_ have smaller band gaps than the end members GaN (3.4 eV) and ZnO (3.2 eV).^5^ The quantum efficiency of the system was later optimized by calcination at 873 K, which resulted in an efficiency of 5.9% at *x* = 0.18.^6^ Subsequently, several studies reported improvements in the photocatalytic activity of the system.^6–9^

Density functional theory (DFT) studies predicted the solid solution has a minimum band gap of 2.4 eV at *x* ∼0.5.^10^ However, achieving this stoichiometry was a non-trivial task for ambient pressure synthesis due to the reduction of Zn^2+^ and its subsequent evaporation at high temperatures.^10–12^ To resolve this challenge, Chen *et al.* synthesized (GaN)_1–*x*_(ZnO)_*x*_ with *x* > 0.3 using high pressure synthesis at around 5 GPa and 1000 °C.^11^ Indeed, as suggested by theory, they observed that the largest visible light absorption occurs at *x* = 0.5, although the exact band gap determination and photocatalytic activity studies were not performed.^11^ It is important to note that later studies reported syntheses under ambient pressure conditions^12,13^ of compositions with even higher ZnO concentrations of up to *x* = 0.9 with their optical properties measured, which are compared with our results.

## Experimental

### GaN synthesis

GaN reagent was synthesized by the ammonolysis of powdered Ga_2_O_3_ (Alfa Aesar, 99.9%). About 0.25 g each of Ga_2_O_3_ powder was placed in two fused silica boats, which were then placed inside a quartz tube (*∅* = 20 mm) going through a tube furnace so that the boats straddle the hot spot of the furnace. Ammonia (Praxair Inc., 99.99%) was passed over the boats at a rate of ∼600 mL min^−1^ while the temperature was raised to 950 °C at a rate of 40 °C min^−1^ and left for 3 hours before quenching. The product was then recovered, ground, and the process was repeated. Completion of the reaction was confirmed by X-ray powder diffraction.

### High pressure synthesis of (GaN)_1–*x*_(ZnO)_*x*_ solid solution

All high pressure reactions were conducted on a ∼ 1 g scale, with a piston-cylinder apparatus and 19 mm diameter cylindrical talc pressure cell. The talc sleeve encircles a cylindrical graphite resistive heater, into which the reactant powder was loaded, capped with two BN disks, and centred within the cell by two pyrophyllite spacers. Before synthesis, the spacers were dried at 1000 °C for 20 minutes. A Pt/Pt90–Rh10 thermocouple was placed in contact with the BN disk to monitor the sample temperature.

Dried powdered reagents ZnO (Aldrich, 99.99%) and GaN were combined in stoichiometric ratios depending on the target composition and ground intimately for 30 minutes. Once loaded, the cell was pressurized to a maximum of 1 GPa. It was then heated, first at 200 °C min^−1^ up to 1000 °C, then slowed to 100 °C min^−1^ up to 1100 °C, and finally 50 °C min^−1^ until the maximum temperature reached; between 1150–1200 °C. The maximum temperature was held for 45 minutes, before being quenched to room temperature, followed by slow decompression over an hour. Samples were recovered as dense sintered pellets, which were cleaned with sandpaper to remove any residual graphite, and then ground into fine powders.

### Compositional analysis by X-ray powder diffraction

X-ray diffraction patterns of the high pressure reaction products were taken on a Rigaku Ultima IV diffractometer (Cu K_α_*λ* = 1.54059 Å, 20–120° 2*θ*, 0.02° step size, 1.5 s per step) in Bragg–Brentano geometry with a D-tex Ultra solid-state detector. A quantitative Rietveld analysis to determine the weight percentages, unit cell volumes and the lattice parameters of all the products was conducted with the program TOPAS-Academic v5.0© 1998-2012 by Alan Coelho (see ESI†).

### Optical properties

Ultraviolet-visible diffuse reflectance data were collected on a Thermo Evolution 300 spectrometer at a range of 300–900 nm. BaSO_4_ was used as the 100% reflectance standard.

### Photocatalytic activity

Photocatalytic water splitting reaction under visible light illumination was carried out in a customized reactor equipped with a quartz window. The reactor was connected to a closed gas circulation and evacuation system. For each experiment, 100 mg sample was dispersed in 180 mL DI water. The system was then purged with ultra-high-purity (UHP) grade Argon gas under stirring for 30 minutes in dark, followed by a degassing procedure. Afterward, 0.8 bar UHP Argon gas was introduced to the system as carrier gas. The suspension was then illuminated by a 300W Xe lamp (Newport, Model 66984) which was equipped with a high pass cutoff filter (>420 nm, Hoya, L42) to eliminate UV light and a 10 cm water filter (>800 nm) to eliminate IR irradiation. The gas products were quantified by using an online gas chromatography (Agilent, 7890A) which was equipped with a thermal conductivity detector (TCD) and a 5 Å molecular sieve column.

### X-ray photoelectron spectroscopy

The lab based ambient pressure XPS experiments were performed on the SPECS Near Ambient Pressure XPS system equipped with a dual anode (Al Ka/Mg Kα) XR-50 NAP X-ray source and a Phobos 150 differentially pumped hemispherical electron analyzer. The (GaN)_1–*x*_(ZnO)_*x*_ with *x* = 0.53 was taken for XPS analysis before and after the photocatalytic activity. The powder is pressed into Al sheets (10 mm × 10 mm × 2 mm) at 2 tons pressure and attached onto the specs stainless steel sample plate with conductive carbon tape. The C 1s photoemission line for adventitious carbon (284.7 eV) was used for the energy calibration.

### ICP-OES analysis

The experiment was conducted on the solution after a run of 6 hour photocatalytic activity testing with an Thermo Sciences iCAP 6300 radial view Inductively Coupled Plasma-Optical Emission Spectrometer (ICP-OES). It measures the elemental concentration of fluid samples. Standardization is achieved by the dilution of 10 000 ppm standards obtained from Spex Certiprep in dilute high purity HNO_3_ that is matrix matched to the liquid that samples are dissolved in. Zn and Ga standards were prepared at concentrations of 0, 1, 4, 20, 100 ppm by gravimetric methods and a calibration curve was generated prior to the analysis of samples. Measurements of instrument baseline and the aforementioned standards allow us to establish that the detection limit for Zn and Ga are 0.05–0.1 ppm and 0.5–2 ppm, respectively.

## Results and discussion

### Compositional analysis

**Fig. 1:**
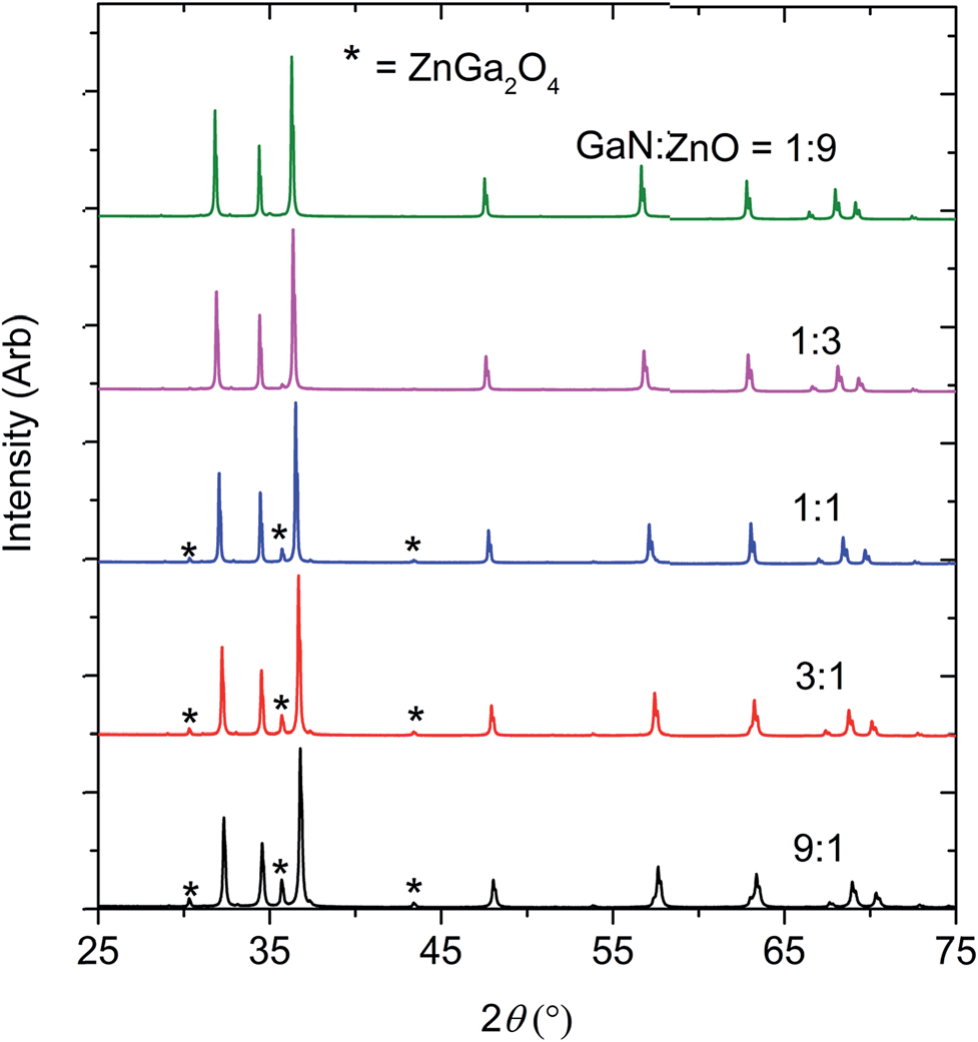
The X-ray diffraction patterns of the products obtained by reacting GaN and ZnO at 1 GPa,1150–1200 °C.

The optimum synthetic temperature range of 1150 °C to 1200 °C, where ZnO completely reacted without reduction, had been determined by several trial experiments. Indeed, Zn metal was present only at composition *x* = 0.51, in an amount less than 0.5 wt%. All samples contained spinel-type ZnGa_2_O_4_ as a second phase, presumed to arise from the reaction between ZnO and Ga_2_O_3_, which may form from partial oxidation of GaN by O_2_ in the reaction environment^11^ (see Fig. 1 and Table 1). Chen *et al.* previously observed ZnGa_2_O_4_ impurity forming in their high pressure syntheses.^11^

**Table 1:** Quantitative Rietveld refinement results of high pressure reaction products, (GaN)_1–*x*_(ZnO)_*x*_ cell parameters and final estimated compositions

GaN : ZnO molar ratios of reactants	Weight percentage of		Lattice parameters of (GaN)_1–*x*_(ZnO)_*x*_ solid solution (wurtzite, *P*6_3_*mc*)		Unit cell volume of (GaN)_1–*x*_(ZnO)_*x*_ (wurtzite, *P*6_3_*mc*)	*R*_wp_ (%)	*R*_exp_ (%)	Estimated composition of (GaN)_1–*x*_(ZnO)_*x*_ phase (*x*) in the product
	(GaN)_1–*x*_(ZnO)_*x*_	ZnGa_2_O_4_	*a* (Å)	*c*(Å)				
9 : 1	87.25(5)	12.75(5)	3.19673(3)	5.18998(6)	45.931(1)	7.00	3.18	0.07
3 : 1	89.58(5)	10.42(5)	3.20721(2)	5.19614(4)	46.288(1)	6.94	3.11	0.24
1 : 1^a^	92.75(4)	6.96(4)	3.22432(1)	5.20620(6)	46.873(1)	6.25	2.83	0.51
1 : 3	98.15 (2)	1.85(2)	3.24060(1)	5.21359(2)	47.415(1)	6.27	1.62	0.76
1 : 9	99.48(3)	0.52(3)	3.24844(2)	5.21665(3)	47.673(1)	10.85	1.68	0.90

**Reference materials**								
ZnO^25^	—	—	3.249	5.198	47.62	—	—	—
GaN^26^	—	—	3.186	5.181	45.73	—	—	—

a This product contains 0.291 wt% Zn (see ESI).

The formation of ZnGa_2_O_4_ causes the final composition of (GaN)_1–*x*_(ZnO)_*x*_ to vary slightly from that expected from the ratio of GaN : ZnO reagents. Determination of the composition *x* from the refinement is difficult since the X-ray scattering contrast between Ga^3+^ and Zn^2+^ and between N^3−^ and O^2−^ is small. Since GaN and ZnO have very close molecular masses (∼3% difference), an approximation of the value of *x* in (GaN)_1–*x*_(ZnO)_*x*_ was adjusted from the stoichiometry of the starting materials using the weight percent of the secondary phases (see Table 1).

**Fig. 2:**
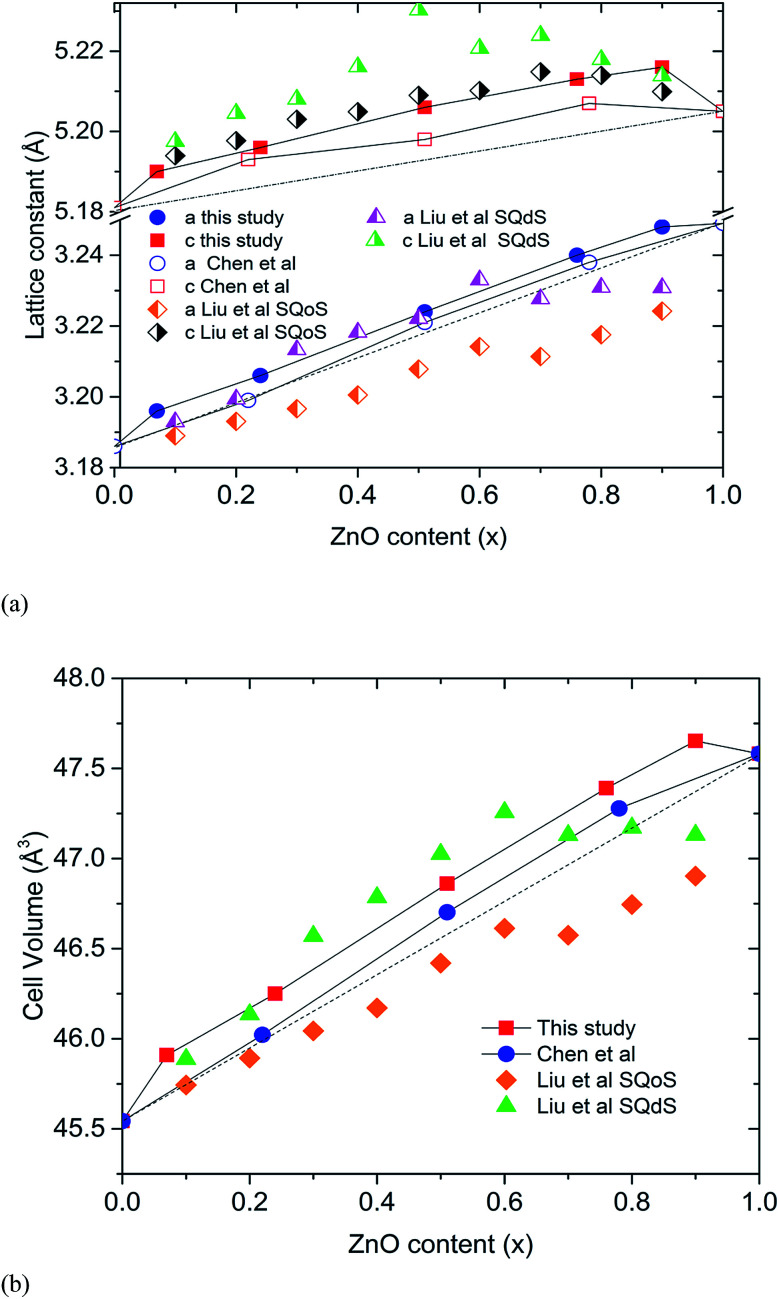
The lattice parameters calculated for high pressure synthesized (GaN)_1–*x*_(ZnO)_*x*_ (top) and cell volumes (bottom) with the values predicted by Liu *et al.* Errors are smaller than markers. Dashed lines represent ideal variation of lattice parameters.

The closed reaction environment of the high pressure system produces relatively pure samples, even for Zn-rich members of the solid solution. Chen *et al.* observed that the lattice parameters of their (GaN)_1–*x*_(ZnO)_*x*_ deviates from the linear dependence on composition expected for Vegard's law, showing an upward bowing trend.^11^ Lattice parameters calculated for our samples show an even higher degree of bowing (Fig. 2). It is theorized that the bowing depends on the degree of disorder and decreases when short-range order (SRO) is present.^10,14^ The SRO in (GaN)_1–*x*_(ZnO)_*x*_ is predicted to occur due to ZnO and GaN clustering driven by the preference for valance-matched Zn–O and Ga–N pairs, and the degree of SRO tends to decrease with increasing synthesis temperature.^10,14^ Recently reported theoretical studies of Liu *et al.* give a comparison of lattice parameters between short-range ordered “special quasi-ordered structure” (SQoS) equilibrated at 1123 K and disordered “special quasi-disordered structure” (SQdS) equilibrated at 20 000 K (see Fig. 2).^15^ The trends of lattice parameters and cell volumes for our samples generally tend to be less than the values predicted for the disordered structure, suggesting some degree of SRO is present. Smaller lattice parameters reported by Chen *et al.* indicate a higher degree of SRO that corresponds to their lower synthesis temperature at 1000 °C.^11^

### Optical properties

**Fig. 3:**
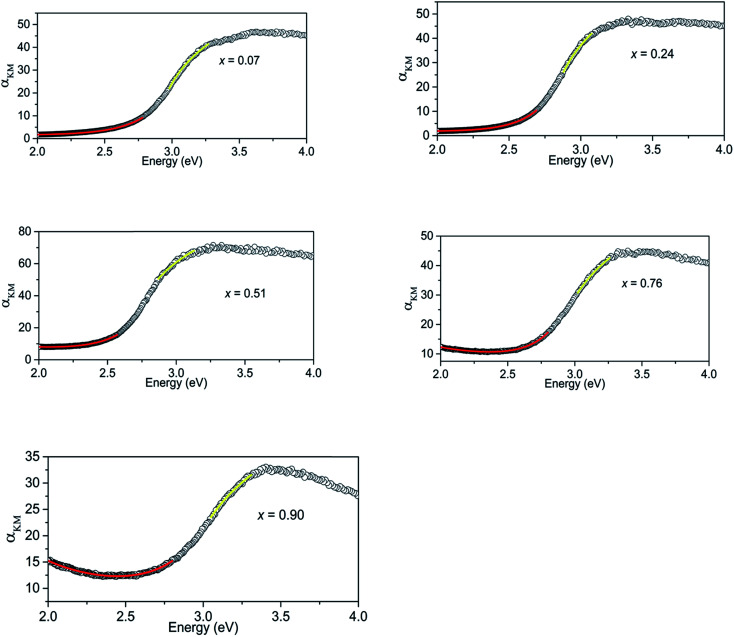
Kubelka–Munk transform of diffuse reflectance data for (GaN)_1–*x*_(ZnO)_*x*_ samples showing regions that show absorption behaviours of direct band gap (yellow) and Urbach tail (red).

The Kubelka–Munk transform of the diffuse reflection data for all the samples are shown in Fig. 3. The highest absorption of light in the visible region is observed at *x* = 0.51. Initial curve fits on our data were made assuming the direct band gap behaviour and take the form,

1 *α*_KM_ = *A*(*E* − *E*_g_)^0.5^/*E*

where *α*_KM_ is the Kubelka–Munk transform of the diffuse reflectance data, *A* is a scaling factor, *E* is the photon energy, and *E*_g_ is the band gap energy. In all the samples, fits done using the formula (1) show direct band gap absorption in the regions where absorption is relatively high (*α*_KM_ > 0.5 *α*_KM, max_) (see the yellow lines in Fig. 3). The calculated band gaps are shown in Table 2. ZnGa_2_O_4_ has a wide band gap of 4.5 eV and is not expected to contribute to absorption in the visible region.^16^

**Table 2:** Calculated band caps and Urbach energies for (GaN)_1–*x*_(ZnO)_*x*_ synthesized at high pressure

Composition (*x*)	Band gap (eV)	Urbach energy (eV)
0.07	2.892(5)	0.216(1)
0.24	2.778(4)	0.179(1)
0.51	2.646(8)	0.203(2)
0.76	2.829(3)	0.185(3)
0.90	2.818(7)	0.27(1)

Absorption of (GaN)_1–*x*_(ZnO)_*x*_ below the band gap can be explained as occurring due to Urbach tail behaviour *α* ∼ exp[(*E* − *E*_g_)/*E*_U_], where *E*_U_ is the Urbach energy, and free carrier absorption *α* ∼ *E*^−3^.^12^ Urbach tail behaviour is explained as the exponential increase in absorption just below band gap energy, occurring due to factors such as impurities, excitons, compositional inhomogeneity and structural disorder.^12,17^ Thus, the regions below band gaps in our data were fitted with both Urbach tail behaviour and free carrier absorption taken in to account. The combined effect takes the form,

2 *α*_KM_ = *A* exp[(*E* − *E*_g_)/*E*_U_] + *BE*^−3^ + *C*

where *A*, *B* and *C* are constants and *E*_g_ can be calculated from eqn (1).^12^ The regions of absorption fitted with eqn (2) are shown with red lines in Fig. 3 and the calculated Urbach energies are shown in Table 2.

**Fig. 4:**
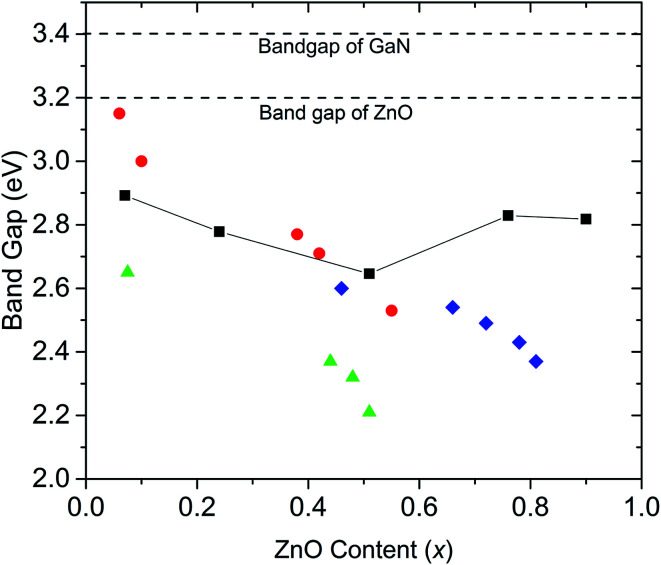
Comparison of band gaps of (GaN)_1–*x*_(ZnO)_*x*_ synthesized at high pressure in this study (black squares, errors are smaller than the symbol), with those of nanorods synthesized by Rienart *et al.*^12^ (red circles), nanoparticles synthesized by Feygenson *et al.*^18^ (green triangles) and (GaN)_1–*x*_(ZnO)_*x*_ synthesized using layered double hydroxide precursors by Wang *et al.*^19^ (blue diamonds). Dashed lines mark the band gap values of GaN and ZnO.

Our experimental results agree with previous DFT studies that predict (GaN)_1–*x*_(ZnO)_*x*_ band gaps follow a downward bowing curve trend with *x*.^10,14^ Jensen *et al.* predicted a minimum band gap of 2.29 eV at *x* = 0.525.^10^ Later, Li *et al.* predicted a minimum band gap to be between 2.5 eV and 2.7 eV at *x* ∼ 0.5, which agrees with our observations.^10,14^ A comparison of band gaps from our study with some other reports are shown in Fig. 4. The smaller band gaps observed on nanoparticulate samples may be due to a higher degree of disorder.^18^

Band gaps reported for samples synthesized at ambient pressure over the entire composition range do not tend to show the downward bowing curve behaviour. For example, Lee *et al.* report onsets of absorption continuously decreasing with increasing Zn content, with onset dropping to 2.2 eV at *x* = 0.87.^13^ However, as shown in this study, band gaps determined by the onset of absorption alone may be underestimated due to Urbach broadening.

Another study reports a similar pattern in materials synthesized from layered double hydroxide precursors with the band gap dropping to 2.37 eV at *x* = 0.81.^19^ Our calculated band gaps tend to agree well with those reported by Reinert *et al.*,^12^ though it should be noted that we used the same method for band gap estimation. This significant variation of band gaps reported by different studies may be explained by the fact that band gaps depend on factors other than the composition including order/disorder and particle size,^14,18,20^ which may vary between materials synthesized under different conditions. Theoretical studies also predict that the band gap of (GaN)_1–*x*_(ZnO)_*x*_ tends to be smaller with increasing disorder.^15^

The Urbach energies estimated for our samples vary between 0.17 eV and 0.27 eV and are larger than those observed before on (GaN)_1–*x*_(ZnO)_*x*_ nanorods (∼0.1 eV) where it was considered to reflect compositional inhomogeneity and/or large concentrations of defects.^12^ As compositional inhomogeneity can be expected due to GaN and ZnO clustering, this can be viewed as another indication of the presence of SRO.

### Photocatalytic activity

**Fig. 5:**
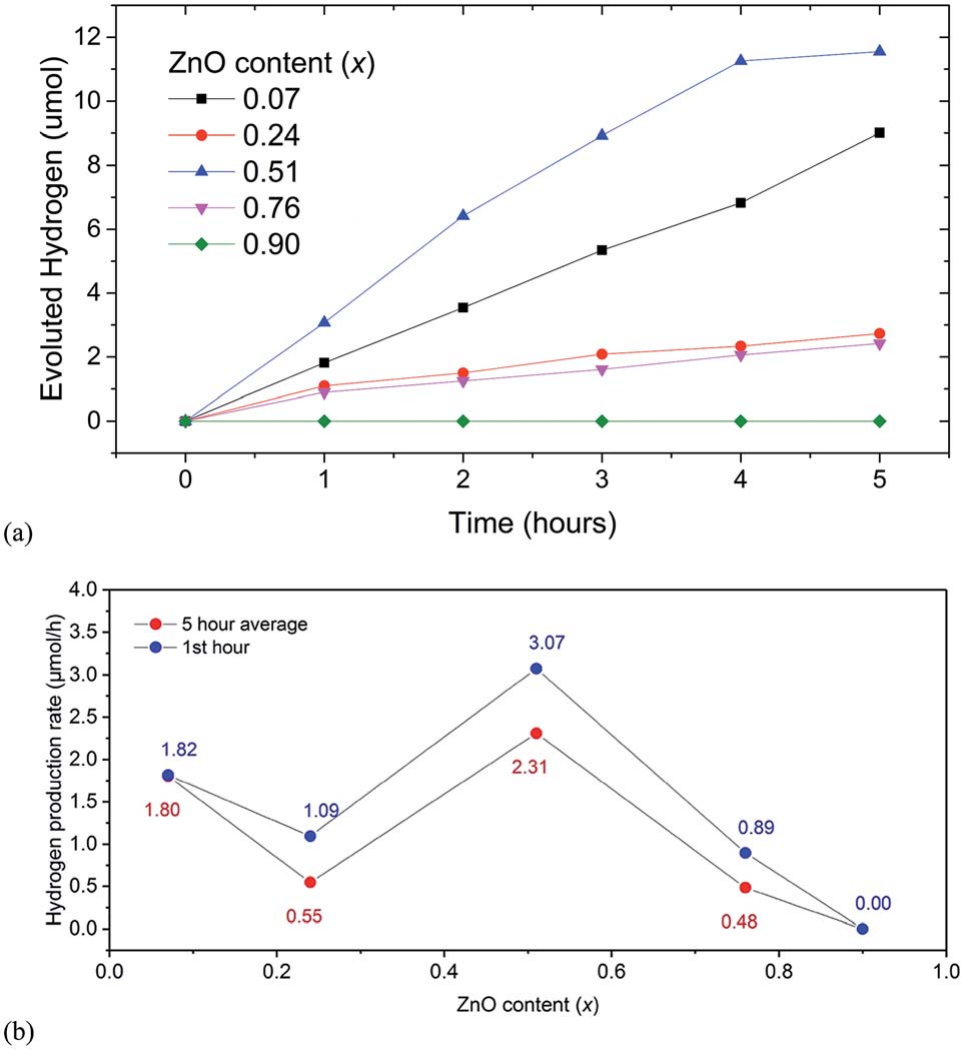
(a) Hydrogen evolution for (GaN)_1–*x*_(ZnO)_*x*_ solid solution members for 5 hours. (b) Hydrogen evolution rates for first hour (blue) and 5 hour average (red).

We observed that the highest average rate of photocatalytic H_2_ evolution of 2.3 μmol h^−1^ under visible light was achieved for samples with *x* = 0.51 without any cocatalysts, sacrificial reagents and pH modifiers (see Fig. 5(a)), while samples with *x* = 0.07, 0.22 and 0.76 showed initial H_2_ evolution rates of 1.8, 1.1, 0.9 μmol h^−1^ respectively. Simultaneous O_2_ evolution was not observed for any sample.

Since UV light was filtered out, ZnGa_2_O_4_, an impurity phase detected in XRD with a wide band gap is not expected to contribute to photocatalytic activity. The sample with *x* = 0.9 showed no activity, although its estimated band gap of 2.82 eV was similar to that of the sample with *x* = 0.76 showing H_2_ evolution. For the sample with *x* = 0.07 the rate of evolution of hydrogen was stable for five hours, while for samples with *x* = 0.24 and 0.76 the rates dropped after one hour and remained constant thereafter. A slight decrease in rate after 4 hours was observed for the sample with *x* = 0.51.

The observed photocatalytic activity shows a complex correlation of H_2_ evolution rate with *x*; the rate decreased from *x* = 0.07 to 0.24 and then increased to a maximum one at 0.51 (see Fig. 5(b)). Similar behaviour is observed in the range *x* = 0.15 to 0.3.^6,21^ It is predicted in the literature and shown in experiments^10,18^ that the extent of disorder correlates to the narrowing of bandgap in general and subsequently the higher photocatalytic activity. The higher extent of the mixing of the GaN and ZnO, we can expect a higher amount of disorder, narrower the bandgap, and higher the photocatalytic activity.

**Fig. 6:**
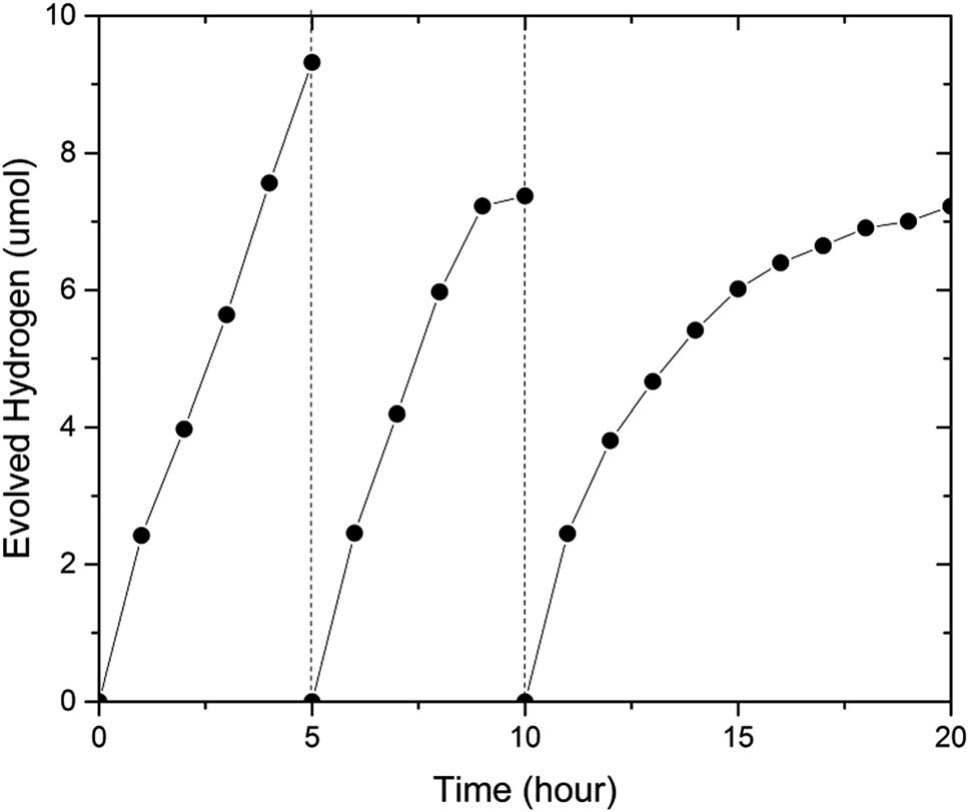
Extended photocatalytic activity of the same sample with composition *x* = 0.53.

To investigate the stability of (GaN)_1–*x*_(ZnO)_*x*_, we carried out an extended photocatalytic activity test for 20 hours, where the sample was periodically evacuated after 5 and 10 hours (see Fig. 6). The sample with *x* = 0.53 utilized in this experiment was synthesized in a multi anvil-press (see ESI†). Importantly, the sample was very stable at the beginning of the experiments. The initial H_2_ evolution rates during each of 5 h period were very similar at 2.42 μmol h^−1^, 2.46 μmol h^−1^ and 2.45 μmol h^−1^ respectively (see Fig. 6). However, the average evolution rate decreased from 1.86 μmol h^−1^ during the first five hour run to 1.47 μmol h^−1^ in the next five hours and finally to 0.722 μmol h^−1^ in the last ten hours. Again, simultaneous O_2_ evolution was not observed.

In comparison, Ohno *et al.* reported (GaN)_1–*x*_(ZnO)_*x*_ activity test periods of 6 months before 50% reduction^22^ when Rh_2-*y*_Cr_*y*_O_3_ was used as a cocatalyst to provide active sites for H_2_ evolution. The significant reduction of activity observed here may be attributed to the fast deterioration of the bare (GaN)_1–*x*_(ZnO)_*x*_ surface as compared to that modified with cocatalyst in Ohno's work. The post-reaction XRD analysis did not find any significant changes in bulk composition of the sample (Fig. 2, ESI†), suggesting that surface dominated de-activation is a plausible explanation. X-ray photoelectron spectroscopy found evidence of oxidation on the Ga sites to GaO_*x*_ supporting this theory (Fig. 3, ESI†). No Zn or Ga was detected in the solution after reaction when analyzed by ICP-OES.

The original work on (GaN)_1–*x*_(ZnO)_*x*_ solid solution system published by Maeda *et al.* used RuO_2_ as a co-catalyst for the overall water splitting.^5^ Subsequent surface modification with Rh and Cr mixed oxide cocatalyst has produced the highest rate of H_2_ evolution for (GaN)_1–*x*_(ZnO)_*x*_.^9,23,24^ Notably, the photocatalytic activities of (GaN)_1–*x*_(ZnO)_*x*_ prepared by the above mentioned authors were negligible in the absence of cocatalysts,^21^ whereas our results clearly show that the catalysts produced at high pressure have measurable activity. Further, the photocatalytic H_2_ evolution activity observed in this study was achieved without sacrificial reagents and pH modifiers, which is remarkable. We anticipate cocatalyst loading on high pressure (GaN)_1–*x*_(ZnO)_*x*_ will further improve stability, and the rate of H_2_ production.

## Conclusions

Members of the (GaN)_1–*x*_(ZnO)_*x*_ solid solution, synthesized up to *x* = 0.9 at *p* = 1 GPa and 1150 > *T* < 1200 °C, were used without surface modification to photocatalytically evolve H_2_ under visible light. High pressure synthesis promoted a more complete solid-state reaction and allows tuning of band gaps and photocatalytic activity for the entire composition range. In agreement with theoretical predictions, lattice parameters and band gaps deviated from Vegard's law, showing an upward and downward bowing trend respectively, with *x*. The smallest band gap of 2.65 eV and largest average photocatalytic H_2_ evolution activity of 2.3 μmol h^−1^ was observed at *x* = 0.51. Reduction of activity was observed over 20 hours presumably due to the deterioration of active surface sites for H_2_ evolution as evidenced by the XPS measurements.

## Conflicts of interest

There are no conflicts to declare.

## Supplementary Material

RA-008-C7RA08509E-s001
