# Supplementary material for: Unexpected visible light driven photocatalytic activity without cocatalysts and sacrificial reagents from a (GaN)1–x(ZnO)x solid solution synthesized at high pressure over the entire composition range
Source: RSC Adv. 2018 Feb 27;8(16):8976–82. doi: 10.1039/c7ra08509e (PMC9078601; doi:10.1039/c7ra08509e)
Supplement: RA-008-C7RA08509E-s001 [file RA-008-C7RA08509E-s001.pdf]

Electronic Supplementary Material (ESI) for RSC Advances.
This journal is © The Royal Society of Chemistry 2018

Electronic Supplementary Information (ESI) for RSC Advances
This journal is © The Royal Society of Chemistry 2017

Unexpected visible light driven photocatalytic activity without cocatalysts and sacrificial reagents from the $(GaN)_{1-x}(ZnO)_x$ solid solution synthesized at high pressure in the entire composition range

Quantitative Rietveld analysis of high pressure synthesis products

High pressure reaction between GaN and ZnO mainly generated wurtzite phase $(GaN)_{1-x}(ZnO)_x$ solid solution along with minor impurities spinel $ZnGa_2O_4$ and hexagonal Zn metal. A quantitative Rietveld analysis to determine the weight percentages, unit cell volumes and the lattice parameters of all phases was conducted with rietveld refinement program TOPAS-Academic v5.0 © 1998-2012 by Alan Coelho. Refinement models were taken from ICSD database; hexagonal space group $P6_3mc$ (#186) was used as a model for $(GaN)_{1-x}(ZnO)_x$ solid solution (ICSD collection code 153304)[1], cubic space group *Fd-3m* (# 227) for $ZnGa_2O_4$ (ICSD collection code 290667)[2] and hexagonal space group $P6_3/mmc$ (# 194) for Zn (ICSD collection code 421014)[3].

The following parameters were refined; background (Chebyshev polynomials with 9 terms), zero error, scale, phase fractions and lattice parameters from all three phases. Each of the $(GaN)_{1-x}(ZnO)_x$ solid solution phase models were refined with the modified Thompson-Cox-Hastings pseudo-voigt profile function with 4 refined terms (U, V, W & Y) and the same values were applied to minor impurity phases as well. B_{eq} and fractional occupancies were fixed at values from the literature.

Table 1: Quantitative Rietveld analysis of high pressure synthesis products

GaN:ZnO molar ratios of reactants	Estimated composition of $(GaN)_{1-x}(ZnO)_x$ phase in the product (x)	Refinement Parameters				Product Phases	Weight Percent	Volume ([Å]^3)	Lattice Parameters	
		R_{exp} (%)	R_p (%)	R_{wp} (%)	GOF				a [Å]	c[Å]
9:1	0.07	3.18	5.42	7.00	2.20	$(GaN)_{1-x}(ZnO)_x$	87.25(5)	45.931(1)	3.19673(3)	5.18998(6)
						$ZnGa_2O_4$	12.75(5)	579.88(3)	8.3390(1)	-
						Zn	-	-	-	-
3:1	0.24	3.11	5.55	6.94	2.23	$(GaN)_{1-x}(ZnO)_x$	89.58(5)	46.288(1)	3.20721(2)	5.19614(4)
						$ZnGa_2O_4$	10.42(5)	579.87(2)	8.3390(1)	-
						Zn	-	-	-	-
1:1	0.51	2.83	4.95	6.25	2.21	$(GaN)_{1-x}(ZnO)_x$	92.75(4)	46.873(1)	3.22432(1)	5.20620(2)
						$ZnGa_2O_4$	6.96(4)	579.48(2)	8.33706(8)	-
						Zn	0.29(2)	30.81(1)	2.6532(3)	5.055(1)
1:3	0.76	1.62	4.93	6.27	3.87	$(GaN)_{1-x}(ZnO)_x$	98.15(2)	47.415(1)	3.24060(1)	5.21359(2)
						$ZnGa_2O_4$	1.85(2)	579.63(3)	8.3378(2)	-
						Zn	-	-	-	-
1:9	0.90	1.68	8.53	10.85	6.47	$(GaN)_{1-x}(ZnO)_x$	99.48(3)	47.673(1)	3.24844(2)	5.21665(3)
						$ZnGa_2O_4$	0.52(3)	579.6(2)	8.3380(7)	-
						Zn	-	-	-	-

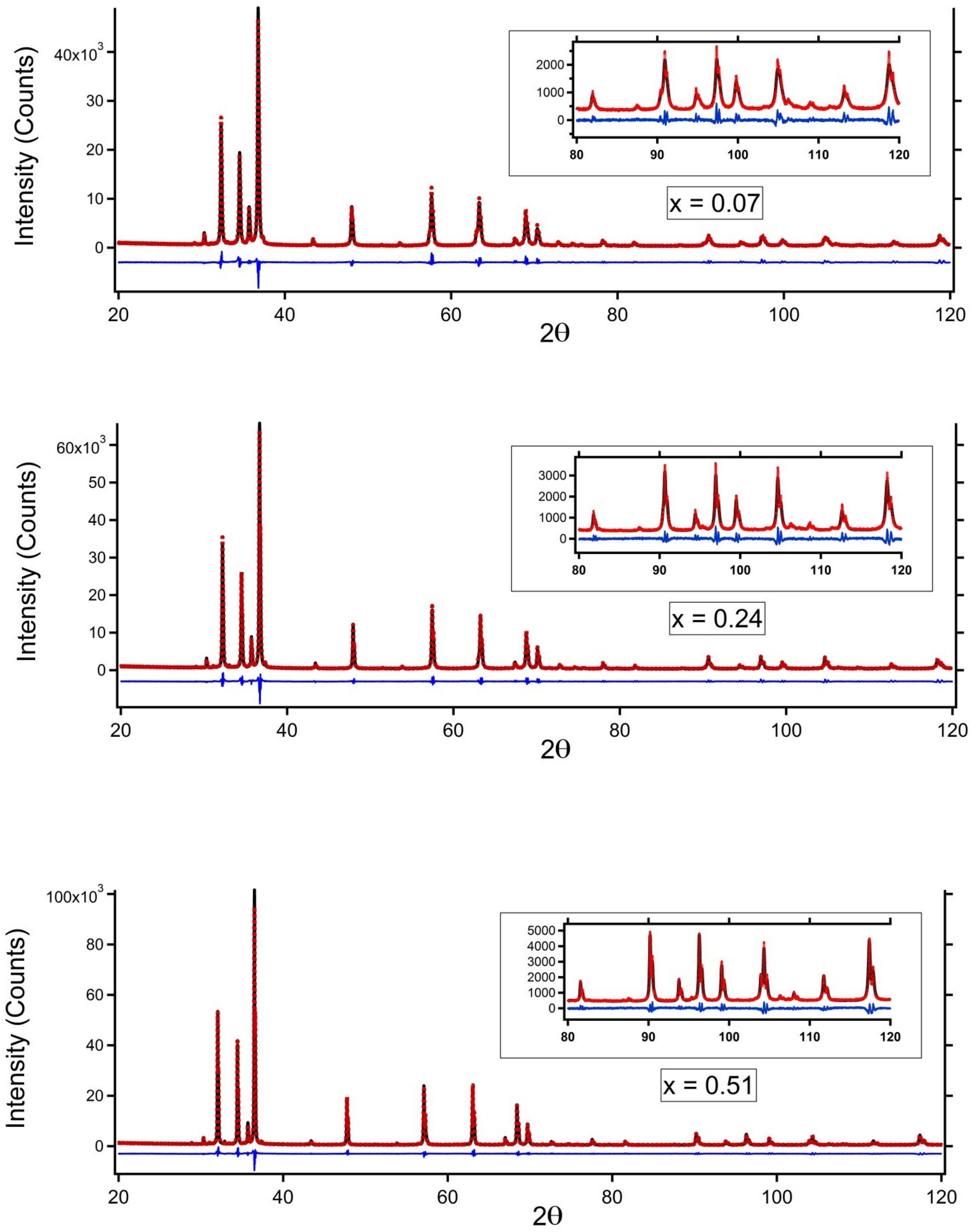
Intensity (Counts)
40x10^3
30
20
10
0
20
40
60
80
100
120
2θ
2000
1000
0
80
90
100
110
120
x = 0.07
Intensity (Counts)
60x10^3
50
40
30
20
10
0
2θ
3000
2000
1000
x = 0.24
Intensity (Counts)
100x10^3
80
60
40
20
0
2θ
5000
4000
3000
2000
1000
x = 0.51

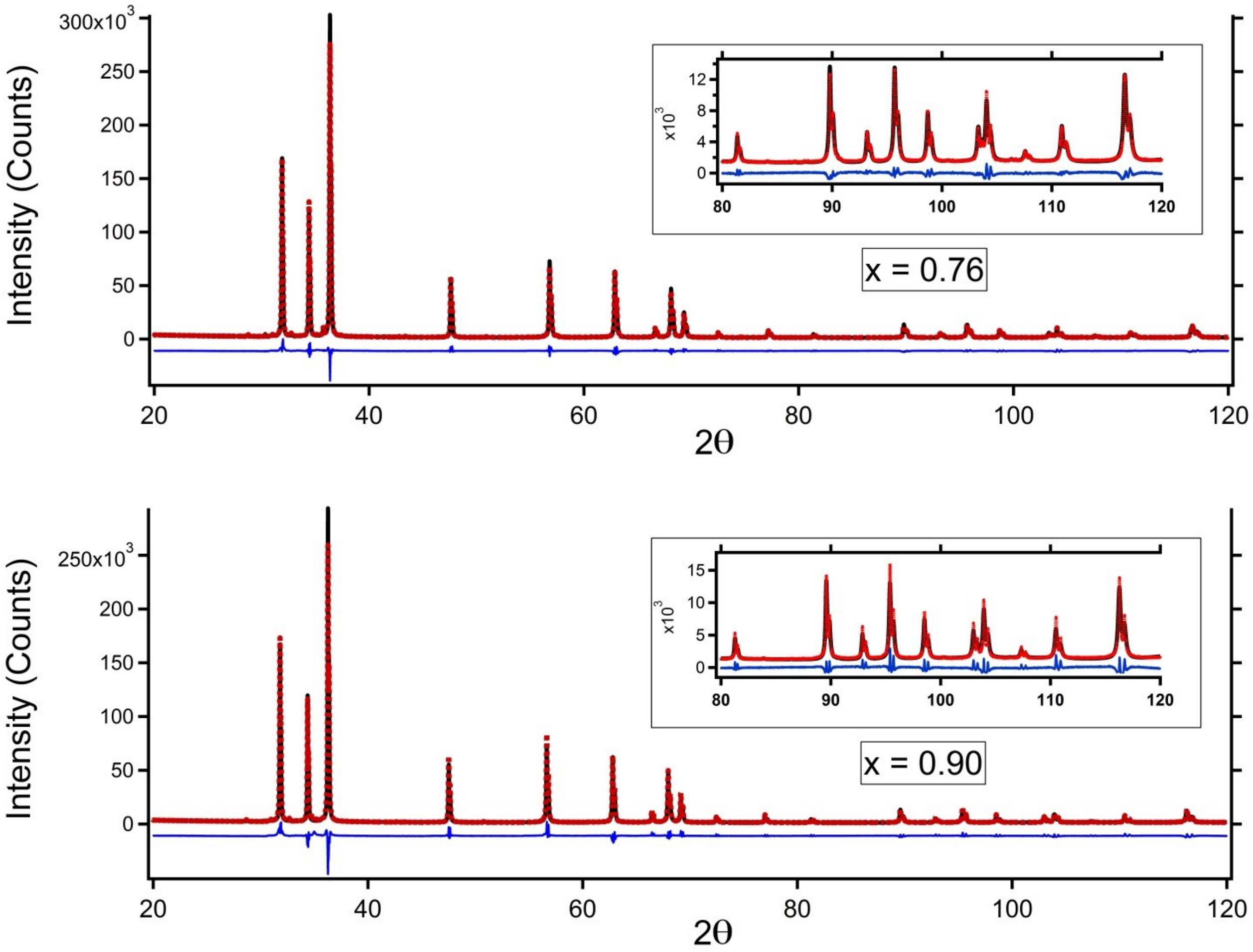

Figure 1: Rietveld refinement patterns of $(GaN)_{(1-x)(}ZnO)_x$ solid solution samples and impurities; black(calculated), red(observed) and blue (difference)

Synthesis of $(GaN)_{1-x}(ZnO)_x$ solid solution ($x = 0.53$) for the extended photocatalytic activity test

The sample was synthesized using a 2000 ton split-sphere multianvil apparatus (USSA-2000). A standard 18/12 cell assembly was used which has been described in detail before[4]. Dried powdered reagents ZnO and GaN were combined in equimolar ratios and ground intimately for 30 mins and around 0.5 g of the sample was sealed in a Platinum capsule and loaded into the cell. Once loaded, the cell was pressurized to a maximum of 2 GPa over 2 hours and then heated. First at 200 °C/min up to 1000 °C, then slowed to 100 °C/min up to 1100 °C and finally 50 °C/min until the maximum temperature reach between 1150 °C - 1200 °C. The maximum temperature was held for 45 mins, before being quenched to room temperature followed by slow decompression over 1 hour. $ZnGa_2O_4$ impurity was present at 15.71% which was increased to 16% after the extended photocatalytic activity test. No other impurities were present.

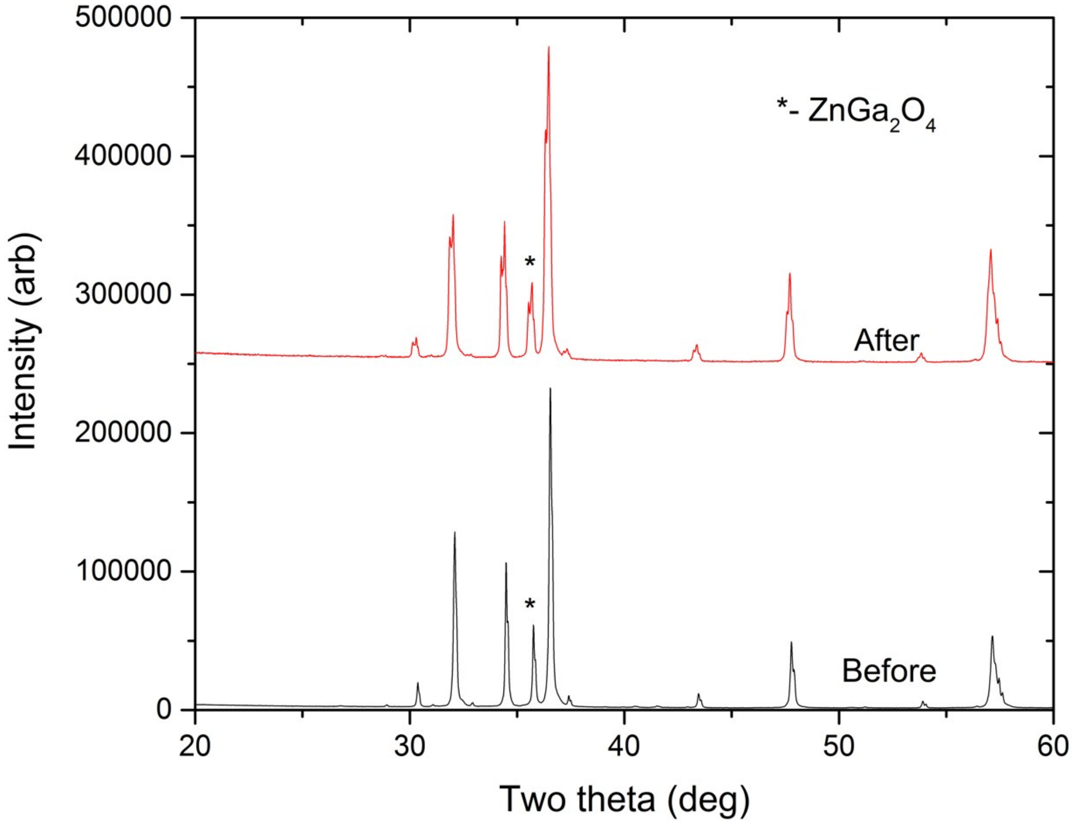

Figure 2: The X-ray diffraction pattern of the $(GaN)_{1-x}(ZnO)_x$ solid solution (x = 0.53) sample before and after the photocatalytic activity test

X-ray photoelectron spectroscopy done on fresh and spent $(GaN)_{1-x}(ZnO)_x$ catalysts

To understand what's happening to the catalysts before and after the photocatalytic activity in water, an XPS analysis was conducted on fresh and spent catalysts. Figure 3 shows the Zn 2p and Ga 2p of the fresh and spent catalysts. Compared to the fresh catalyst, the spent catalyst appears to contain an appreciable amount of oxidized Ga (denounced GaO_x). The Zn 2p of the spent catalyst has a lower binding energy feature at 1020.3 eV, which is even lower than that of metallic Zn (1021.7 eV) and is attributed to e^- dense ZnO.

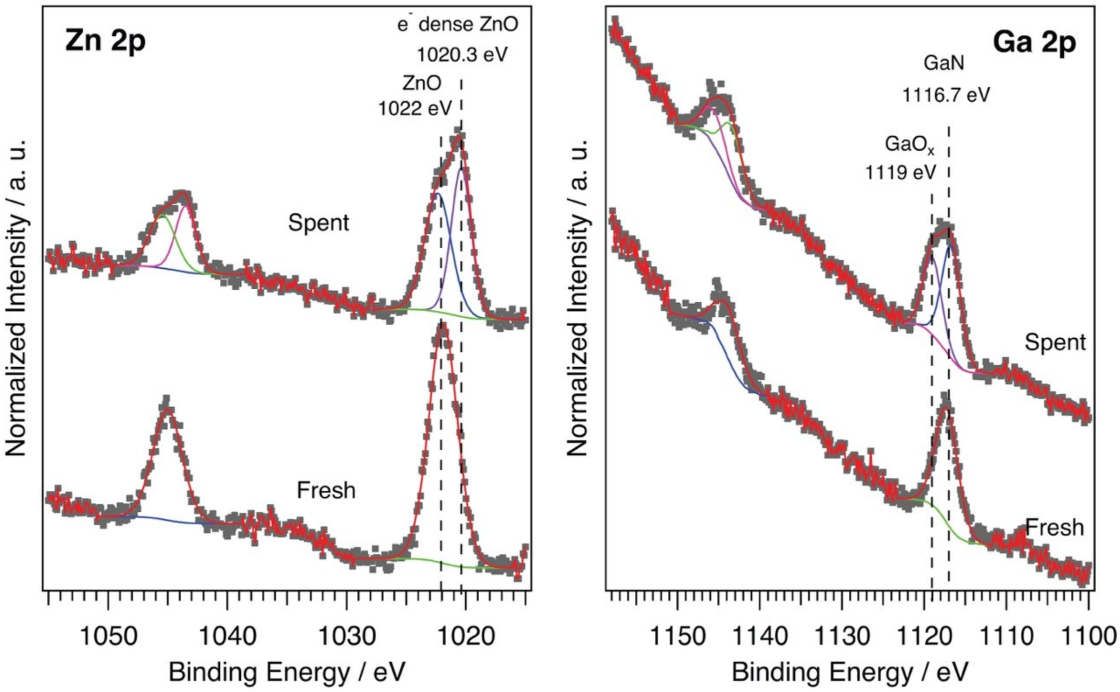

Figure 3. Zn 2p (left) and Ga 2p (right) of $(GaN)_{1-x}(ZnO)_x$ before and after reaction.

Figure 4 shows the C 1s, O 1s and N 1s of the two catalysts. The N 1s signal is considerably low and is not able to produce a reliable fit. It is clear however that N is present in both fresh and spent catalysts. The C 1s region is dominated by adventitious carbon (expected) and likely carbonates at 288-289 eV. While the feature assigned as carbonate is present in the fresh catalyst, it significantly increases in the spent catalyst. The O 1s shows a similar trend. The peak at 532 eV is assigned as carbonates, however some is due to GaO_x as their contributions can not be distinguished. The peak at ~530 eV is due to ZnO and the peak at 533 eV in the spent catalyst is assigned as hydroxyls adsorbed on the surface after the reaction.

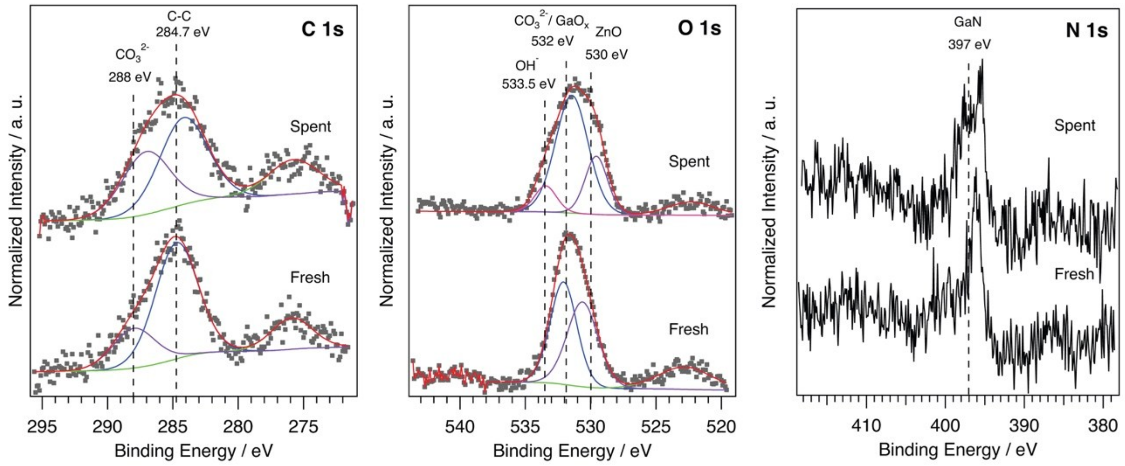

Figure 4. C 1s (left), O 1s (middle) and N 1s (right) of $(GaN)_{1-x}(ZnO)_x$ before and after reaction.

Figure 5 shows the LMM Auger transition for Zn and Ga. The bulk of Zn is ZnO in both the fresh and spent catalyst. The Ga LMM is mainly GaN, but a shoulder at lower kinetic energy appears in the spent catalyst, which is likely due to a more oxidized form of Ga; This correlates with the added features in the Ga 2p.

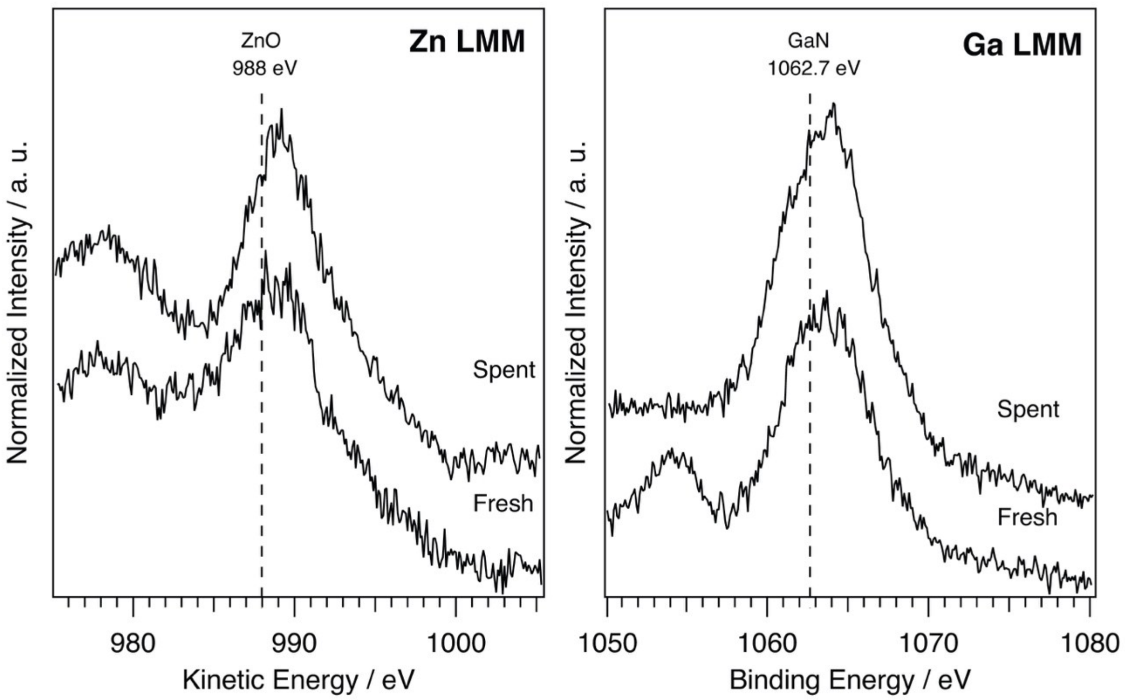

Figure 5. Zn LMM (left) and Ga LMM (right) of $(GaN)_{1-x}(ZnO)_x$ before and after reaction.

References

1 M. Yashima, K. Maeda, K. Teramura, T. Takata and K. Domen, *Chem. Phys. Lett.*, 2005, 416, 225–228.

2 F. Zerarga, A. Bouhemadou, R. Khenata and S. Bin-Omran, *Solid State Sci.*, 2011, 13, 1638–1648.

3 J. Nuss, U. Wedig, A. Kirfel and M. Jansen, *Zeitschrift fur Anorg. und Allg. Chemie*, 2010, 636, 309–313.

4 E. Stoyanov, U. Haussermann and K. Leinenweber, *High Press. Res.*, 2010, 30, 175–189.
